# CCL5 and CD11b-fibrin interactions regulate microglia migration and morphology in hyperinflammatory experimental cerebral malaria

**DOI:** 10.1186/s12974-026-03969-y

**Published:** 2026-07-24

**Authors:** Nadia D. Domingo, Olivia D. Solomon, Paula Villarreal, Florentin Aussenac, Difernando Vanegas, Mark Joseph Endrino, Andrew S. Mendiola, Lucinda Puebla-Clark, Komi Gbedande, Astrid E. Cardona, Katerina Akassoglou, Matthew J. Flick, Gracie Vargas, Robin Stephens

**Affiliations:** 1 Center for Immunity and Inflammation, Rutgers New Jersey Medical School, Newark, NJ USA; 2 Department of Internal Medicine, Division of Infectious Diseases, University of Texas Medical Branch, Galveston, TX USA; 3 Institute of Translational Sciences, Human Pathophysiology/Translational Medicine, University of Texas Medical Branch, Galveston, TX USA; 4 Department of Neurobiology, University of Texas Medical Branch, Galveston, TX USA; 5 Department of Molecular Microbiology and Immunology, The University of Texas at San Antonio, San Antonio, TX USA; 6 Center for Neurovascular Brain Immunology at Gladstone and UCSF, San Francisco, CA USA; 7 Gladstone Institutes of Neurological Disease, San Francisco, CA USA; 8 Weill Institute for Neurosciences, Department of Neurology, University of California, San Francisco, CA USA; 9 Department of Pathology and Laboratory Medicine, University of North Carolina at Chapel Hill, Chapel Hill, NC USA; 10 UNC Blood Research Center, University of North Carolina at Chapel Hill, Chapel Hill, NC USA; 11 Department of Regional Health Research, University of Southern Denmark, Esbjerg, Denmark; 12 Department of Pharmacology, Physiology and Neuroscience, Rutgers New Jersey Medical School, Newark, NJ USA; 13 Present Address: Department of Biology, Prairie View A&M University, Prairie View, TX USA

**Keywords:** Cerebral malaria, Mice, Fibrinogen, Microglia, Chemokines, Neuroinflammation

## Abstract

**Background:**

Microgliosis and severe coagulation, including fibrinogen deposition, are features of both experimental and human cerebral malaria (CM), a lethal disease. Vascular-associated microglia migrate to coagulated cerebral vessels containing inflammatory monocytes and T cells in experimental CM. We previously showed that microglial depletion exacerbates coagulation and disease severity, including hypothermia, while anticoagulant treatment reduces microgliosis and prevents mortality. These data suggest an overall protective effect of microglia on eCM, and indicate a link between microgliosis, hypothermia, and coagulation. Therefore, mechanisms of migration and activation of microglia, T cells, and monocytes were studied in relation to the role of fibrin(ogen) in eCM.

**Methods:**

Using both *Plasmodium berghei* ANKA infection or experimental CM, and *P. chabaudi* infection of IL-10-deficient mice (IL-10 KO), which causes a hyperinflammatory response including neuropathology, intravital two-photon microscopy and flow cytometry, were used to test patterns and mechanisms of migration. In vivo methods included intranasal administration of CCL5 receptor antagonist Met-CCL5; systemic integrin-blocking antibodies and mutant animals (IL-10 KO, ICAM1 KO), and anticoagulant treatment. Clotting-deficient mice (*Fga* KO, *Fib*^AEK^) were tested for the absence of fibrinogen in clotting, while fibrinogen γ-chain mutation (Fibγ^390–396 A^) mice and intranasal administration of a fibrinogen γ-derived inhibitory peptide (Fibrin γ^377–395^), which disrupts CD11b-fibrin interactions, assessed the role of CD11b-fibrin interactions in microglial activation in eCM.

**Results:**

Intraluminal adhesion and crawling of CCR2^RFP+^ cells, including T cells and inflammatory monocytes, was observed in cerebral vessels; however, there was no evidence that brain adherence of T cells or monocytes depends on classical adhesion molecules or coagulation. Intranasal administration of met-CCL5 reduced both microglial recruitment to vessels and CD8 T cell adherence and fibrin(ogen) deposition. Despite the previously published protective effects of the anticoagulant drug in IL-10 KO, clotting-deficient mice showed no change in *P. berghei* ANKA mortality. However, disruption of fibrin-CD11b interactions by both genetic and peptide inhibition led to exacerbated hypothermia in both experimental CM models. Reduced microglial hypertrophy also occurred in Fibrin γ^377–395^ peptide-treated IL-10 KO mice infected with *P. chabaudi*.

**Conclusions:**

These data support our previous study suggesting that microglia are involved in the regulation of hypothermia in eCM and reveal CCL5 and fibrin-CD11b signaling in microglia as key molecular pathways modulating neuroinflammation in malaria.

**Supplementary Information:**

The online version contains supplementary material available at 10.1186/s12974-026-03969-y.

## Introduction

Malaria remains one of the most devastating infectious diseases worldwide. Despite progress in parasite and vector control measures and the introduction of a malaria vaccine, the global burden continues to rise, with an estimated 263 million cases and 610,000 deaths in 2024, 95% of which occurred in Africa [1]. Cerebral malaria (CM) is the most lethal manifestation of severe malaria and primarily affects children. CM is best defined as retinal changes in combination with loss of consciousness from parasite infection, and can include seizures, coma, and a high rate of neurological sequelae among survivors [2, 3]. Disease severity is strongly influenced by host genetics, including polymorphisms in cytokine genes, such as tumor necrosis factor (*TNF)*,* IL12*, and *IL10* [4–7]. During infection with *Plasmodium spp.*, a delicate balance between pro- and anti-inflammatory cytokines is required to limit both parasitemia and immunopathology. The pro-inflammatory cytokines Tumor Necrosis Factor (TNF) and its family member, Lymphotoxin (LT), are critical in the pathogenesis of eCM. Lymphotoxin-α, produced by a radiation-resistant cell population, is the principal mediator of *P. berghei* ANKA-induced eCM, while TNF is dispensable for mortality and endothelial activation [8]. IL-10 and TGFβ are the primary regulatory cytokines controlling lethal pathology in eCM and are genetically linked to severity in CM [8, 9]. In human CM, a ratio of serum IL-10 to TNF of less than one is a risk factor for both CM and severe anemia [10]. An antibody that recognizes both TNF and LT is protective in IL-10 KO mice infected with *P. chabaudi* and prevents gliosis [9, 11]. The significance of regulatory cytokines in malaria pathology has been highlighted through infection of IL-10-deficient mice (IL-10 KO) with *Plasmodium chabaudi* AS. This infection results in a mild disease in wild-type mice; however, in the absence of the regulatory cytokine IL-10, infection with *P. chabaudi* triggers a hyperinflammatory response characterized by cerebral pathology, including edema, hemorrhage, and neurological dysfunction [12]. This model of hyperinflammatory malaria captures key aspects of inflammation-driven vascular and neural pathology, providing a powerful system to dissect cytokine-mediated mechanisms of CM. Notably, cytokines contribute to endothelial activation, establishing a prothrombotic and inflammatory environment in both CM and experimental cerebral malaria (eCM) that contributes to coagulation, vascular congestion, and blood–brain barrier (BBB) breakdown [13, 14]. The mechanism by which this inflammatory vascular environment is transmitted to the brain parenchyma in *P. falciparum*, which shows microgliosis [15], is not fully understood. In IL-10 KO mice infected with *P. chabaudi*, we found that fibrin(ogen) is increased in the liver and deposited in the brain vasculature, occluding blood flow [16]. Neutralization of TNF in this model eliminates occlusive fibrin(ogen) deposition, establishing that inflammation drives the coagulopathy in hyperinflammatory eCM. Importantly, anticoagulant treatment reduces the early mortality of eCM as well as microglial activation, underscoring a mechanistic link between inflammation-induced coagulation and neuroinflammation [16]. Studies of multiple coagulation factors in human and mouse malaria indicate a role for coagulation in the severity of cerebral malaria. In addition to fibrin deposition blocking and slowing down blood flow during CM, clots are required for the repair of microhemorrhage, a common feature of mouse and human CM. Systemic levels of clotting factors are elevated in most CM patients, as the syndrome includes coagulopathy in most patients. Overt disseminated intravascular coagulation correlates with increased mortality [13]. In general, von Willebrand Factor (vWF), Tissue Factor (TF), platelets, and fibrinogen bind at sites of endothelial cell activation or death. Deficiencies in coagulation factors, including platelets and vWF affect both parasitemia and disease severity, which are also linked through the involvement of endothelial protein C receptor in both parasite sequestration and regulation of coagulation [17, 18].

Microglia, the resident macrophages of the brain, constantly monitor the microenvironment and respond rapidly to vascular and inflammatory cues. In brains from children who had an autopsy after dying of CM, microglia have been detected expressing MHCII as well as the early marker of activation, MRP8 [19]. Only a few microglia expressing CD68, a marker of phagocytic vesicles, have been detected in human CM [15, 19], and no systematic study of these cells has been conducted to date. Microglia can contribute to neuroinflammation; however, their primary role is to maintain brain homeostasis, for example, by phagocytosis of particulates in the brain parenchyma. New roles for microglia are still being discovered; for example, a series of recent papers demonstrates a role for microglia in the regulation of capillary diameter and patency [20–22]. We previously observed an increase in activated microglia lining cerebral vessels near sites of fibrin(ogen) deposition, and that microglia depletion worsens disease in *P. chabaudi-*infected IL-10 KO, indicating that microglia play a protective role in eCM [22]. Microglial depletion significantly increased vascular coagulation, suggesting direct or indirect interactions of microglia with the coagulation cascade [22]. Consistent with this, microglia detect vascular damage through leakage of fibrin(ogen) and cytokines in neurodegeneration and -inflammation [23, 24]. Indeed, microglia engulf fluorescent fibrin(ogen) as early as day 3 post-infection in *P. chabaudi* infection of IL-10 KO [22]. Therefore, microglia activation in response to vascular damage is likely to be an early step in the neuropathology of cerebral malaria. However, microglia interaction mechanisms with the coagulation cascade have not been previously reported in eCM.

The molecular signals directing leukocyte adhesion within the vessels and microglial recruitment to vascular lesions during malaria also remain unclear. The chemokine receptors CXCR3, CCR5, and CCR2 all contribute to eCM; however, specific mechanisms are still being determined [25–27]. We previously reported an increase in the chemokine CCL5 in infected IL-10 KO versus uninfected animals and found this chemokine colocalized with activated microglia and fibrin(ogen) in the brain tissue, suggesting a role of this chemokine in microglia migration to the clotted areas [22]. The cytokine-induced endothelial activation central to much of CM pathogenesis includes the upregulation of Intercellular Adhesion Molecules (ICAM) and Vascular Cellular Adhesion Molecule (VCAM), which are known to promote both the adhesion of immune cells and the sequestration of infected red blood cells (iRBCs) in the brain microvasculature [28, 29].

Here, we investigated how inflammation-induced coagulation and chemokine signaling coordinate microglia migration and activation during hyperinflammatory eCM. We characterized the immune cells adherent within and present in apposition to the cerebral vasculature in the brain parenchyma, including T cells, inflammatory monocytes, and microglia. We also tested the contribution of integrins and chemokines to their accumulation. We identified CCL5 as a key factor in recruiting microglia and CD8 T cells to the vasculature. Using fibrinogen-mutant mice and intranasal fibrin-CD11b blocking peptides, we also demonstrated that fibrin(ogen) activates microglia through CD11b. Furthermore, we demonstrate that the interaction of CD11b with fibrin(ogen) does not affect survival or parasitemia but plays a role in regulating body temperature during infection, suggesting another specific function that may be attributed to microglia.

## Methods

### Mice

Experimental animals included several genetically modified mouse strains for infection by *P. chabaudi*. IL-10-deficient (B6.129P2-IL10^tm1Cgn/J /J, JAX #002251) mice were used alongside IL-10 KO reporter animals, aiding fluorescence-based identification of microglia and monocytes (IL-10 KO CX3CR1^GFP^ CCR2^RFP^) [22, 30]. To investigate combined genetic effects, IL-10 KO mice were crossed with ICAM-1 KO (*Icam1*^*tm1Jcgr*^/J, JAX # 005983). Fibrinogen mutant mice—including Fibγ^390–396A^ [31], *Fib*^AEK^ [32], and fibrinogen alpha knockout (*Fga* KO) [33] on the C57BL/6 background. All mice were bred either at the University of Texas Medical Branch Animal Resource Center or at the Rutgers New Jersey Medical School animal facility. Experimental cohorts consisted of male and female mice, aged 8–12 weeks and weighing at least 20 g at the time of infection. Animals were maintained under specific pathogen-free conditions with ad libitum access to food and water.

### In vivo studies

To study the role of coagulation in cell adhesion, we treated mice with Low Molecular Weight Heparin (LMWH, Enoxaparin sodium, Sanofi-Aventis, Bridgewater, NJ) at 1 mg/Kg twice a day, intraperitoneally (i.p.) at 4, 5, and 6 days post-infection (dpi). The control group was treated with sterile saline. To assess the role of adhesion molecules, animals were treated with 200 µg/Kg/day Anti-VCAM1 (CD106, #BE0027 BioXcell, Lebanon, NH) or isotype control, IgG1 anti-horseradish peroxidase (HRPN, #BE0088 BioXcell) [34], at 4 and 6 dpi. To inhibit ICAM-2, 3 mg/kg/day of anti-ICAM-2 (clone 3C4 M/C2/4, #553325, BD Pharmingen), or isotype rat IgG2a Kappa (BD Pharmingen # 553927) was given to infected mice once a day via i.p. injection at 6 and 7 dpi. Platelet depletion was performed using monoclonal rat anti-mouse GPIb-α (CD42b, #R300, Emfret Analytics, Würzburg, Germany), while the control group was treated with a polyclonal non-immune rat IgG antibody control (#C301; Emfret Analytics, Eibelstadt, Germany). Antibodies were diluted in 200 µl sterile phosphate-buffered saline (PBS) and injected i.p. into the mice at 2 µg/g body weight [35, 36]. In CD11b blocking peptide experiments, mice were treated once a day with Fibrin γ^*377–395*^ peptide (30 µg in 10 µl) [37], scrambled peptide, or saline via the intranasal route daily from 4 to 8 dpi. Alexa Fluor 647-conjugated fibrinogen (1.5 mg/mL; Invitrogen, Thermo Fisher Scientific, Waltham, MA) was administered intravenously (i.v., 100 µL of 1.5 mg/mL) at 48 and 24 h before the end of the experiment. Met-CCL5 (#278-RN, R&D Systems, Mineapolis, MN) was given intranasally (1 µg/kg/day) daily from 3 to 6 dpi [38, 39].

### Parasite and infection

Frozen stocks of *P. chabaudi chabaudi* AS (originally from Jean Langhorne, Crick Institute, London, UK via Patrick Duffy, NIAID), and *P. berghei* ANKA iRBCs (Clone cl15cy1, stock #MRA-871) from BEI Resources Repository, NIH, kept in liquid nitrogen, were quickly thawed and injected by i.p. route. At the time of infection, blood from mice infected with frozen stock was used as donors before the peak of infection and diluted in Krebs–Ringer bicarbonate buffer (Sigma-Aldrich, St. Louis, MO) and normal saline (Sigma-Aldrich, St. Louis, MO) to deliver 1 × 10^5^ iRBCs *P. chabaudi* or 1 × 10^6^ *P. berghei* ANKA, in 200 µl. For all experiments, thin blood smears were collected from the tail at regular intervals (5–7 dpi) to monitor peripheral parasitemia. Peripheral parasitemia was quantified by staining smears with Giemsa stain (Ricca Chemical Company, Arlington, TX) and counting iRBCs on a Nikon Eclipse 80i light microscope, as in [9].

### Pathology measurements

Mice were assessed daily for internal body temperature starting at day 5 post-infection (dpi) using a stainless-steel rectal probe and digital rodent model thermometer (BIO-TK8851; Braintree Scientific, Braintree, MA). The probe was sanitized after each mouse with CaviCide and dried before reuse. Weight measured on a portable balance (OHAUS, Parsippany, NJ) was recorded in grams. Starting at 5 dpi, daily behavioral assessments were performed on all animals. An abbreviated version of the modified SmithKline Beecham, Harwell Imperial College, Royal London Hospital Phenotype Assessment (SHIRPA) protocol [40] was used to measure general health, baseline behaviors, and sensory and neurological function. The abbreviated SHIRPA involves nine semi-quantitative tests selected for their significantly different outcomes between *P. chabaudi-*infected C57BL/6 and IL-10 KO [16, 40]. The expected score for a healthy, uninfected IL-10 KO mouse ranges from 21 to 23. In contrast, a score below 16 before day 9 in IL-10 KO mice infected with *P. chabaudi* is statistically correlated with the death of eCM within the next 12 h [11]. The assessment takes approximately 5 min per animal. First, the animal is placed in a 600 ml glass beaker on top of the cage grid for 3 min. The following behaviors are recorded without disturbing the animal: Body Position (BP) ranged from 0 (completely flat) to 5 (repeated vertical leaping), while Spontaneous Activity (SA) ranged from 0 (None, resting) to 4 (extremely vigorous, rapid/dart movement). From the viewing enclosure, the animal is transferred onto the cage floor without being handled. Palpebral Closure (PC), ranging from 0 (eyes closed) to 2 (eyes wide open); Gait (G), 0 (incapacity) to 3 (normal); and Tail Elevation (TE) during forward motion from 0 (dragging) to 2 (elevated) are measured. After this, Touch Escape (TEs) is assessed with a finger stroke, and ranges from 0 (no response) to 3 (vigorous escape response to approach); qualitative Grip Strength (GS) was measured by lowering the animal and allowing it to grip a metal grid; then, a gentle horizontal backward pull was applied. The score ranged from 0 (no grip) to 4 (unusually effective grip). Finally, during supine restraint, the animal is scruffed firmly, and Heart Rate (HR) and Righting Reflex (RR) are recorded. Heart rate is felt by palpation through the sternum, scored from 0 (slow, bradycardia) to 2 (fast, tachycardia). Just after that, the animal is flicked back into the open arena. We measured the Righting Reflex (RR); the observed landing position was scored from 0 (fails to right itself when placed on its back) to 3 (no impairment). During the behavioral assessment, animals were randomly assigned to treatment groups and co-housed within the same cages to avoid cage-specific effects. Each mouse was identified by an ear-punch, and behavioral assessments were conducted based on this identifier rather than treatment group.

### Thin-skull cranial preparation

For intravital microscopy, experimental mice were anesthetized with a mixture of ketamine/dexmedetomidine (0.1 ml/10 g) and kept on a heating pad, with tail and toe pinch responses monitored. The head was immobilized with a stereotactic device (Stereotaxic 520, Steoelting, Wood Dale, IL) during the thin-skull cranial procedure. Ophthalmic ointment (#NDC11695-6832-1, Covetrus, Dublin, OH) was applied to both eyes to prevent drying. The surgical area was cleaned with 70% alcohol and wiped using room-temperature PBS. A hair depilatory (Nair, Ewing, NJ) was applied, and a midline incision was made to expose the skull. A metal (zinc) washer, with a diameter of 1/2 inch (229489, Home Depot, Atlanta, GA), was applied to the head of the mouse using Cyanoacrylate (#EM-02, Starbond, Los Angeles, CA) to maintain liquid tension during intravital imaging. The fascia was scraped away to reveal the skull entirely. A high-speed dental drill (#NC9010016, Braintree, MA) with a round carbide bur (Meisinger, Centennial, CO) was used to thin the skull just below the coronal suture and bregma and parallel to the sagittal suture. The drill was operated in gentle circular motions to remove a portion of the spongy bone until pial blood vessels could be visualized under a stereotactic microscope (SMZ445, Nikon, Brighton, MI), and the remaining bone showed flexibility to gentle pressure from the drill tip. PBS or Artificial Cerebrospinal Fluid (ACSF) (#597316, Harvard Apparatus, Holliston, MA), warmed in an incubator, was frequently reapplied during pauses in drilling to prevent drying, overheating, and to remove debris. If bleeding occurred due to rupture of vessels in the spongy bone, gel foam (#09-0315-08, Pfizer, New York, NY) or bone wax (#W31G, Ethicon, Cincinnati, OH) was applied to impacted areas, and fresh PBS or ACSF was applied. Before placing the mouse under the objective of the upright two-photon fluorescence microscope (Prairie Technologies Ultima IV, Bruker), the surgical area was thoroughly cleaned, and fresh PBS was applied to aid visualization under the microscope.

### Intravital Microscopy (IVM) by two-photon excited fluorescence microscopy

Intravital microscopy (IVM) of the subpial superficial cortex was performed by two-photon fluorescence excitation microscopy using an Ultima IV upright multiphoton microscope (Bruker, Middleton, WI) using a Mai Tai femtosecond laser for fluorescence excitation primarily at 800 nm (Spectra Physics, Santa Clara, CA). Anesthetized mice with a thinned-skull window preparation were placed prone on a heating pad beneath a 40 × 0.8 N.A. water immersion objective (NIR Apo, 0.80 W, DIC N2, WD 3.5) focused on the superficial cortex. This lens provided a field-of-view of 321 × 321 μm. Two- channel acquisitions for GFP and RFP were performed using a fluorescence detection cube with a 575 nm dichroic mirror and a bandpass filter centered at 604 nm (45 nm bandwidth) and 525 nm (70 nm bandwidth). For the acquisition of Alexa 647, an additional cube that included the bandwidth of 660/40m (dichroic, 585 nm) was used. Time series (T-series) to visualize immune cell dynamics and fibrin(ogen) uptake by microglia in vivo were obtained at an interval of 3 ms between frames with a frame size of 512 × 512 pixels. Z-stacks with thicknesses of 50 to 200 μm were obtained with a typical step size of 1.0 μm and an image frame size of 1024 × 1024 pixels. Four to six sites were imaged per mouse. Image files were acquired in 12-bit TIFF format and converted to an 8-bit TIFF format for processing and quantification of features using Fiji-ImageJ (version 2.1.0, Java 1.8.0, NIH) and/or Imaris (Bitplane, version 9.7). For analysis and quantification, 8-bit TIFF images were first background-subtracted using a rolling ball radius of 25 pixels, a median filter (using a two-pixel radius), and despeckled to remove nonspecific speckle noise using the ‘despeckle’ filter. Fiji-ImageJ software was used to complete most measurements as described below for the analysis of images from intravital microscopy. Imaris was used to create 3-dimensional renderings of depth stacks [41].

### Immunofluorescence

For ex vivo studies, right atrial perfusion was performed using ice-cold PBS (20 ml) followed by 4% cold paraformaldehyde (PFA, Sigma) at a rate of 4–7 mL/min using a syringe pump (BS-9000-2, Braintree Scientific). Extracted tissues were then fixed overnight in 4% PFA at 4 °C in a cold room. Whole brains were fixed and then hemisected and subsequently cryoprotected by immersion in 30% sucrose for 3 days in 15 mL conical tubes at 4 °C in a cold room. For cryosection, tissues were embedded in an Optimal Cutting Temperature Compound (OCT 4585, Fisher Healthcare, Houston, TX), snap-frozen in liquid nitrogen until hardened, and stored in a − 80 °C freezer. 30 μm sagittal sections were cut using a cryostat LEICA CM 1950 and allowed to free-float in a 12-well flat-bottom cell culture plate (3513, Corning Inc.) with 1 ml PBS. Tissues were blocked in 5% goat serum (Goat Serum 50062Z, Life Technologies, Frederick, MD) in PBS with Triton-X100 (Sigma) for 1 h at RT. Brain sections were stained for microglia overnight with gentle agitation in a cold room ( 4 °C) using polyclonal anti-Iba-1 (019-19741, Fujifilm Labchem Wako, Osaka, Japan), or rabbit monoclonal anti-TMEM119 (ab209064, ABCAM, Cambridge, UK), at 1/1000 dilution. A secondary antibody to reveal microglia was used at 1:1000 and stained 1 h RT (AF-488 goat anti-rabbit, IgG (H + L) A11008, Invitrogen, Life Technologies, Eugene, OR). We used Tomato lectin (Dylight 594, #L32471, Thermo Fisher Scientific, Waltham, MA) at 1/1000 to stain brain vessels. Brain sections were then washed three times with PBS/Triton X-100 (0.3%) in 15-min intervals with gentle agitation. The sections were lastly washed with PBS and mounted onto glass slides with mounting media (ProLong Gold antifade reagent, Invitrogen, Ref. P36934). Sections were imaged using Leica Stellaris 8 tau-STED confocal microscopy in the Rutgers NJMS Cellular Imaging and Histology Core.

### Analysis of IVM images and immunofluorescence markers

In IVM, microglia were identified based on the CX3CR1^GFP^ signal and morphology. Engulfment of Alexa Fluor-647-fibrinogen conjugate was determined by assessing the colocalization of AF647 signal and microglia (CX3CR1^GFP^). In immunofluorescence, Iba-1 staining was used to identify microglia and confirm characteristics seen by IVM. Image thresholding in Fiji-ImageJ was used to segment individual Iba-1-stained microglia in images for morphological analysis. Microglia Sholl analysis was performed using the ImageJ Sholl Analysis plugin (https://imagej.net/Sholl), in which the first shell was set 10 μm beyond the soma border, and ‘parameters’ set to ‘linear’ with a step size of 5 µm. The parameter, transformation index, reflective of activation-associated morphology, was also applied to image-segmented microglia in Fiji-ImageJ and calculated as microglia perimeter^2^/4π × area^2^ [21, 42].

### 3D and movie image analysis

Image files were acquired in 12-bit TIFF format and processed in an 8-bit TIFF format for various variables using Fiji-ImageJ (version 2.1.0, Java 1.8.0, NIH) and/or Imaris (Bitplane, version 9.7). For analysis and quantification, 8-bit TIFF images were imported and processed using Fiji-ImageJ by applying a background subtraction with a rolling ball radius of 25 pixels, a median filter with a radius of 2 pixels, and processed by the ‘despeckle’ filter to remove any nonspecific speckly noise. Fiji-ImageJ software was used to complete most measurements, as described below, for the analysis of images from intravital microscopy [43, 44]. Imaris (Concord, MA, USA) was used to create 3-dimensional renderings of depth stacks acquired from experiments to determine further spatial distribution, microglia, and morphology [41].

### Flow cytometry of immune cells in the brain

Infected mice were anesthetized with isoflurane (0.25–3% in Oxygen, to effect) and perfused transcardially with 40 mL of ice-cold PBS at a rate of 4–7 mL/min using a syringe pump (BS-9000-2, Braintree Scientific). Brains were collected and placed in ice-cold FACS buffer [PBS containing 2% fetal bovine serum (Sigma-Aldrich) and 0.1% sodium azide (Cat# 7144.8–16, Ricca Chemical, Arlington, TX)]. Single-cell suspensions were prepared by gently homogenizing brain tissue using syringes and filtering the cell suspension through a 70 μm cell strainer into PBS. Mononuclear cells were enriched by density gradient centrifugation on a 30/70% Percoll (Sigma-Aldrich) gradient, and the interphase was collected. Alternatively (Fig. 3), brain single-cell suspensions were prepared using the Adult Brain Dissociation Kit, mouse and rat (Miltenyi Biotec, #130-107-677) according to the manufacturer’s instructions. Cell viability was assessed using Live/Dead Fixable Blue (Ref. L23105A, Invitrogen) and then incubated with anti-mouse CD16/CD32 (clone 2.4G2, Bio X Cell, Lebanon, NH) for 20 min at 4 °C to block Fc receptor-mediated binding. For surface staining, the following antibodies were used (from BioLegend, San Diego, CA unless otherwise indicated): anti-Ly6C-FITC (HK1.4, #128006) or Ly6C-BV421 (HK1.4, #128031); CD3-BV510 (17A2, #100233); BV510 (53 − 6.7, #563068, BD); CD4-PerCP/Cy5.5 (RM4-5, #45-0042-82, Invitrogen); (CD90.2) Thy-1.2-PerCP/eFluor 710 (30-H12, #46-0903-82, Invitrogen, Waltham, MA) or -FITC (30-H12, #11-0903-82, Invitrogen); CD45.2-PE (104, #12-0454-8, Invitrogen) or -BUV395 (104, #564616, BDbiosciences); CD11b-PE/Cy7 (M1/70, #552850, BDbiosciences) or PE/Cy5 (M1/70, #15-0112-83, Invitrogen); MHCII-APC (I-A/I-E, M5/114-15-2, #17-5321-82, Invitrogen). Antibody incubation was for 40 min at 10 °C. Samples were acquired on an LSRII Fortessa flow cytometer (BDbiosciences, San Jose, CA) and analyzed using FlowJo v10.10.0 (BDbiosciences, Ashland, OR). Compensation controls included unstained cells and single-stained splenocytes (anti-CD4 in each fluorochrome).

### Statistics

Groups were compared using either Student’s *t-test* (for two groups) or one-way ANOVA followed by Tukey’s test (more than two groups). Statistical analysis was performed in Prism (version 9.5.1, GraphPad, La Jolla, CA). Statistical significance is indicated as ns (not significant), or p ≦ 0.05 (*), and error bars represent ± SEM. Three to five mice per group were used in these studies. For immunohistochemistry, three to five areas in the cortex and hippocampus were assessed per mouse, and five mice per group.

## Results

### Microglia with internalized fibrinogen and CCR2⁺ leukocytes accumulate around cerebral vasculature during hyperinflammatory eCM

**Fig. 1:**
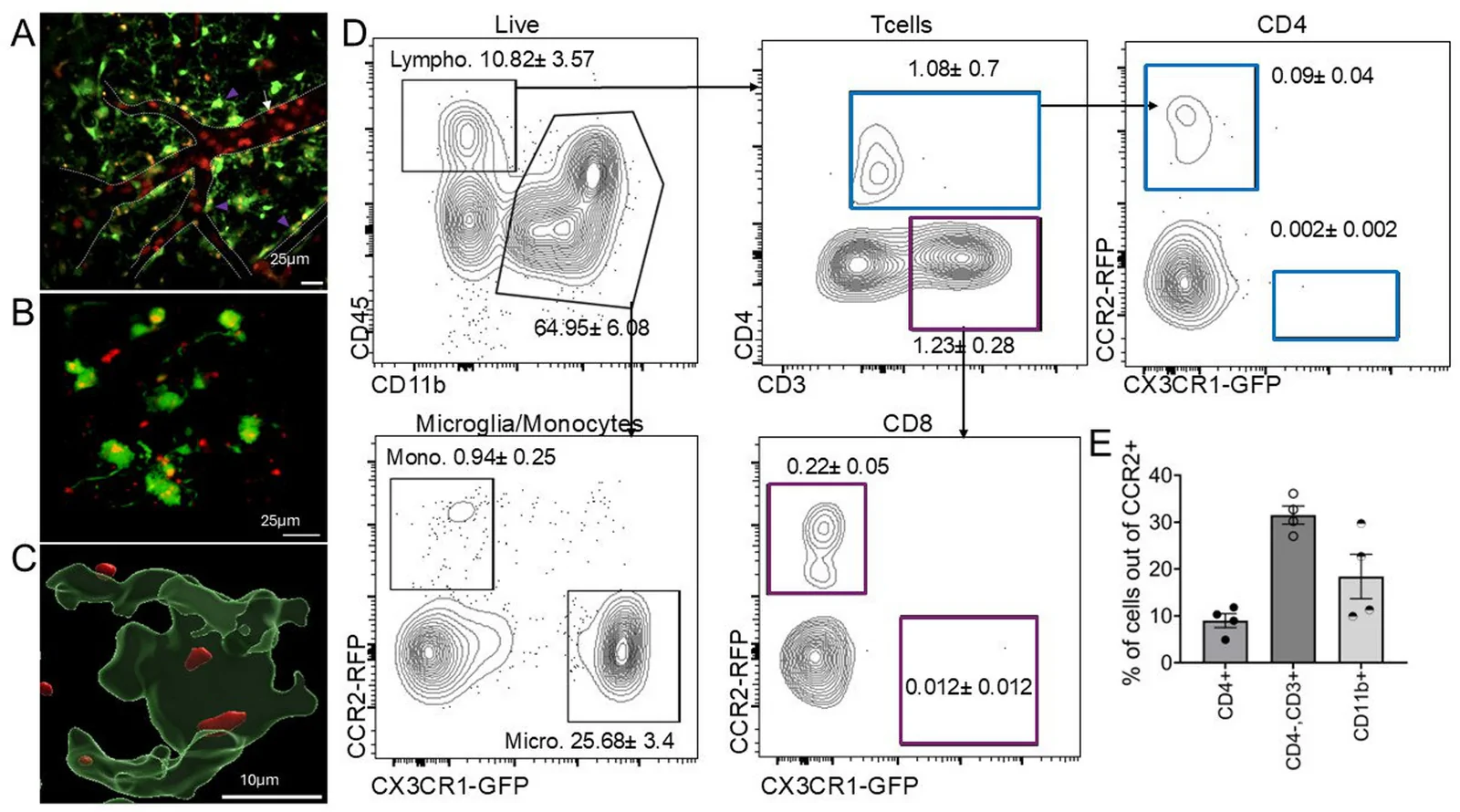
Microglia with internalized fibrin(ogen) and CCR2⁺ leukocytes accumulate around cerebral vasculature during hyperinflammatory eCM. IL-10 KO reporter mice (CX3CR1^GFP^CCR2^RFP^) were infected with *P. chabaudi* and studied 7 dpi. **A-C** Fibrinogen-Alexa Fluor647 was given i.v. 48 and 24 h before sacrifice. CCR2^RFP+^(red) and CX3CR1^GFP+^ (green) cells and fluorescent fibrinogen (AF647, red particles) inside CX3CR1^GFP+^ cells (yellow), and extracellular fibrinogen in the parenchyma (red) are also shown. Intravital two-photon micrographs from a thinned-skull window show **A**) uptake of fibrinogen along the vessels by microglia. The vessel is demarcated using a weak signal in the Alexa Fluor647 channel (white dotted line). **B** Fluorescent fibrinogen within microglia or extracellular, and **C**) an isolated microglia from the image in B shown as a volumetric reconstruction with fluorescent fibrinogen (red) inside microglia (green). **D** Concatenated contour plots showing flow cytometry and average fractions out of live cell gate of brain cells from infected IL-10 KO reporter animals after perfusion. Microglia (CD11b^+^,CX3CR1^GFP+^)and adherent T cells (CD45^hi^CD11b^−^Thy1^+^, CD3^+^CD4^+^ or CD4^−^CD3+^+^(CD8 or NKT), and monocyte-derived cells (CD11b+, CCR2^RFP+^). Numbers on plots are infected group average fraction out of live cell gate +/- SEM (*n* = 3–5)

Our previous imaging and cytometry studies have shown that both T cells and inflammatory monocytes are adherent in the perfused cerebral vasculature of mice with hyperinflammatory eCM (IL-10 KO infected with *P. chabaudi*), and this has been shown in *P. berghei* ANKA infection [11]. Intravital microscopy of *P. chabaudi*-infected reporter (IL-10 KO CX3CR1^GFP+^ CCR2^RFP+^ knock-in) mice, injected with Fibrinogen-AF647 (i.v.), was used to identify potential interactions between microglia, monocytes, vasculature, and fibrin(ogen) in vivo (Fig. 1) [24]. We observed the relative colocalization of microglia with the vasculature in infected IL-10 KO CX3CR1^GFP^CCR2^RFP^ reporter mice during the peak of hyperinflammatory eCM, just before mortality, as ascertained by low scores on the modified SHIRPA behavioral assay. Microglia (CX3CR1^GFP+^) aligned along the parenchymal side of the vascular walls, while CCR2^RFP+^ cells flowed by and accumulated in some locations intravascularly in infected animals 7 dpi (Supplemental Fig. 1). RFP+ cells were observed to be clustered in what have been termed hotspots (Fig. 1A). Intravital imaging also revealed CX3CR1^GFP+^ vascular associated microglia (VAM) next to the vessel demarcated using a weak signal from fibrinogen-Alexa Fluor 647 (Fib-AF647; Fig. 1A, white dotted line), and internalized Fib-AF647 (Fig. 1B). We previously quantified a progressive increase in microglia with internalized fibrin(ogen) between 3 and 7 dpi and showed that over 75% of microglia take up Fib-AF647 by 7 dpi, when given Fib-AF647 i.v. for two days previously [22]. In that study, and also visible here (red), endogenous fibrin(ogen) was also found as isolated puncta within the parenchyma outside of microglia [16]. Volumetric image reconstruction of isolated microglia with co-localized Fib-AF647 illustrates complete internalization of Fib-AF647 in morphologically activated CX3CR1^GFP+^ microglia (Fig. 1C). The uptake of fibrin(ogen) by microglia lining the vasculature over time was also visualized by intravital microscopy of IL-10 KO microglia reporter animals, which were given i.v. Fib-AF647 (Supplemental Fig. 1), and individual microglia can be seen taking up Fib-AF647 (Supplemental Fig. 2).

Flow cytometry was used to identify the immune cell types in the brains of infected animals (Fig. 1D) and to identify the CCR2^RFP+^ cell types seen by microscopy (Fig. 1E). Cells were isolated from the brains of IL-10 KO CX3CR1^GFP^CCR2^RFP^ reporter mice infected with *P. chabaudi*. We report here that overall, lymphocytes (CD45^hi^, CD11b^**−**^) made up 10.82% of the live cell gate in brains of infected reporter mice, including CD4^+^CD3^+^ (1.08% of live) and CD8 + or NKT (1.23.% of live, CD45^hi^CD11b^**−**^CD4^−^CD3^+^). Gating within the CD4 + T cell population showed that 0.09% of live cells were CD4 + CCR2^RFP+^, but very few were CX3CR1^GFP+^. CD8 and NKT cells (CD4-CD3+) CCR2^RFP+^ were 0.22% of live, while CD8 CX3CR1^GFP+^ were 0.012% of live cells, suggesting that most T cells were non-fluorescent. CD11b+ cells contain some CCR2^RFP+^ (0.94% CCR2^RFP+^ CX3CR1^−^ of live), and a majority of CX3CR1-GFP microglia. These flow cytometry data suggest that the cells seen rolling along intravascularly and contributing to vascular occlusion include both Monocyte-derived cells and T cells. To study the red cells seen in microscopy, we gated on live cells in the brain and found 4.29% of live to be CCR2^RFP+^; however as suggested by the above analysis, not all CCR2 + are monocyte-derived (Fig. 1E). We found 8.99% of Live CCR2^RFP+^ cells are CD3 + CD4+ cells, while CD45^hi^CD3^+^CD11b^−^CD4^−^ populations (which include CD8, NKT) represent 31.50%, with 18.40% of CCR2 + live cells expressing the CD11b+ surface marker including monocyte-derived cells.

### Blocking integrins or coagulation did not significantly change the adhesion of T cells or inflammatory monocytes in the brain vessels in hyperinflammatory eCM

**Fig. 2:**
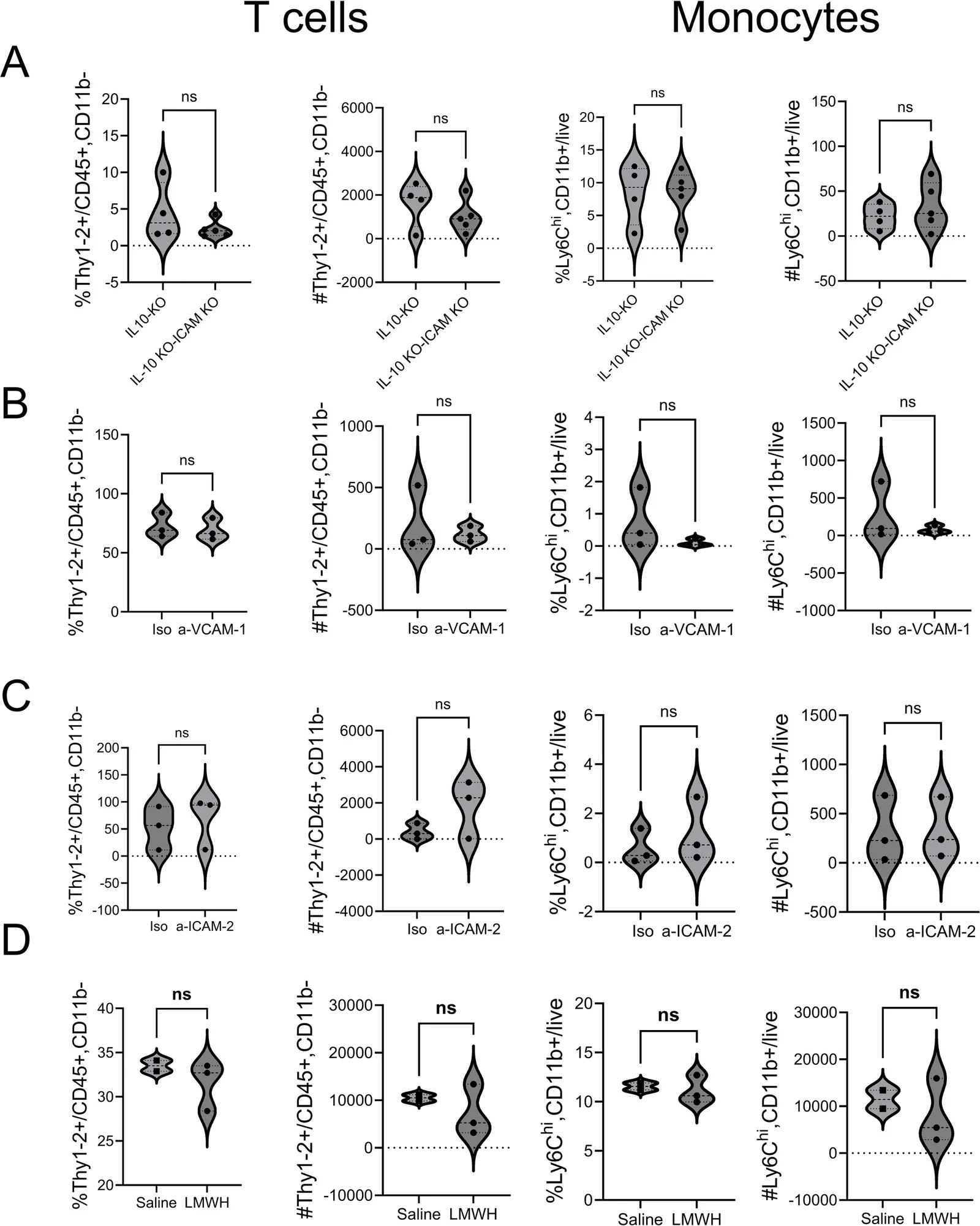
Blocking integrins or coagulation did not significantly change the adhesion of T cells or inflammatory monocytes in the brain vessels in hyperinflammatory eCM. IL-10 KO mice were infected with *P. chabaudi* and perfused brains assayed by flow cytometry 7 dpi. Adherent T cells (CD45^hi^CD11b^−^Thy1^+^) and inflammatory monocytes (CD11b^+^Ly6C^hi^) were quantified and shown in violin plots showing percent and number per brain in (**A**) IL-10 KO compared to IL-10 KO ICAM-1 KO. **B**,** C** Infected IL-10 KO ICAM-1 KO were treated with (**B**) anti-CD106 (VCAM1) or (**C**) anti-CD102 (ICAM2), or an isotype control 4, 6 dpi (i.v.). **D** IL-10 KO mice treated with low molecular weight heparin (LMWH) or saline twice a day on 5, 6 dpi. Each experiment was repeated twice (*n* = 3–5/group). Data are represented as violin plots showing the mean and 95% CI. ns, not significant by unpaired Student’s *t-test*

We previously observed an increase in immune cells in the brain of *P. chabaudi-infected* IL-10 KO mice compared to uninfected mice and infected C56BL/6 mice [11]. As integrins are used by T cells and inflammatory monocytes for firm adherence to endothelial cells, we assessed the roles of ICAM-1 (CD54), VCAM-1 (CD106), and ICAM-2 (CD102), which serve as key binding partners for integrins in leukocyte adherence, for their specific roles in adherence of leukocytes to the vasculature in eCM. In all experiments, mice monitored for clinical signs and parasitemia were perfused on day 7 pi with ice-cold PBS to remove non-adherent cells, and brains were collected for flow cytometry. Gating strategy for identification of inflammatory monocytes and T cells is shown in Supplemental Fig. 3. IL-10 KO ICAM-1 KO animals infected with *P. chabaudi* showed no difference in frequency of brain-adherent T cells (CD45^hi^ Thy1.2^+^) or inflammatory monocytes (CD45^hi^CD11b^+^Ly6C^+^) compared to control IL-10 KO (Fig. 2A). There was reduced *P. chabaudi* parasitemia in IL-10 KO ICAM-1 KO mice compared to IL-10 KO, as previously published in ICAM-1 KO [45]. Though limited cell numbers make quantification unreliable, CD4⁺ and CD8⁺ populations seem to be equally unaffected by the treatments. Of note, one caveat is that the cells termed T cells using Thy1.2+, CD45+,CD11b- may include in the gate NKT cells or innate lymphoid cells that have not been previously studied in experimental CM. To test for potential redundancy for VCAM-1 or ICAM-2 with ICAM-1, IL-10 KO ICAM-1 KO mice were infected with *P. chabaudi* and treated with neutralizing antibodies to VCAM-1 (Fig. 2B) or ICAM-2 (Fig. 2C), which also had no significant effect on T cells or monocytes in the brain of infected animals and did not change the parasitemia. No statistically significant differences in behavior were observed between the groups in these experiments. As we had previously observed leukocytes within fibrin clots by immunofluorescence, an alternate explanation for cellular adherence after perfusion is that the cells are trapped by vascular congestion, including coagulation [11]. Therefore, starting on 5 dpi, we treated mice with Enoxaparin, a low molecular weight heparin (LMWH) that can rescue IL-10 deficient animals from *P. chabaudi-*induced mortality, and tested adherence of leukocytes 7 dpi (Fig. 2D). Though LMWH treatment decreases intravascular fibrinogen deposition [16], LMWH did not change the numbers of adherent T cells or inflammatory monocytes compared to the untreated group. Overall, these data do not provide evidence for roles for classic adhesion molecules or coagulation in leukocyte adhesion in hyperinflammatory eCM.

### Inhibition of CCL5 chemokine receptor binding decreases sickness behavior, vascular-associated microglia, CD8 T cells, and fibrin(ogen) deposition in the brain but not parasitemia

**Fig. 3:**
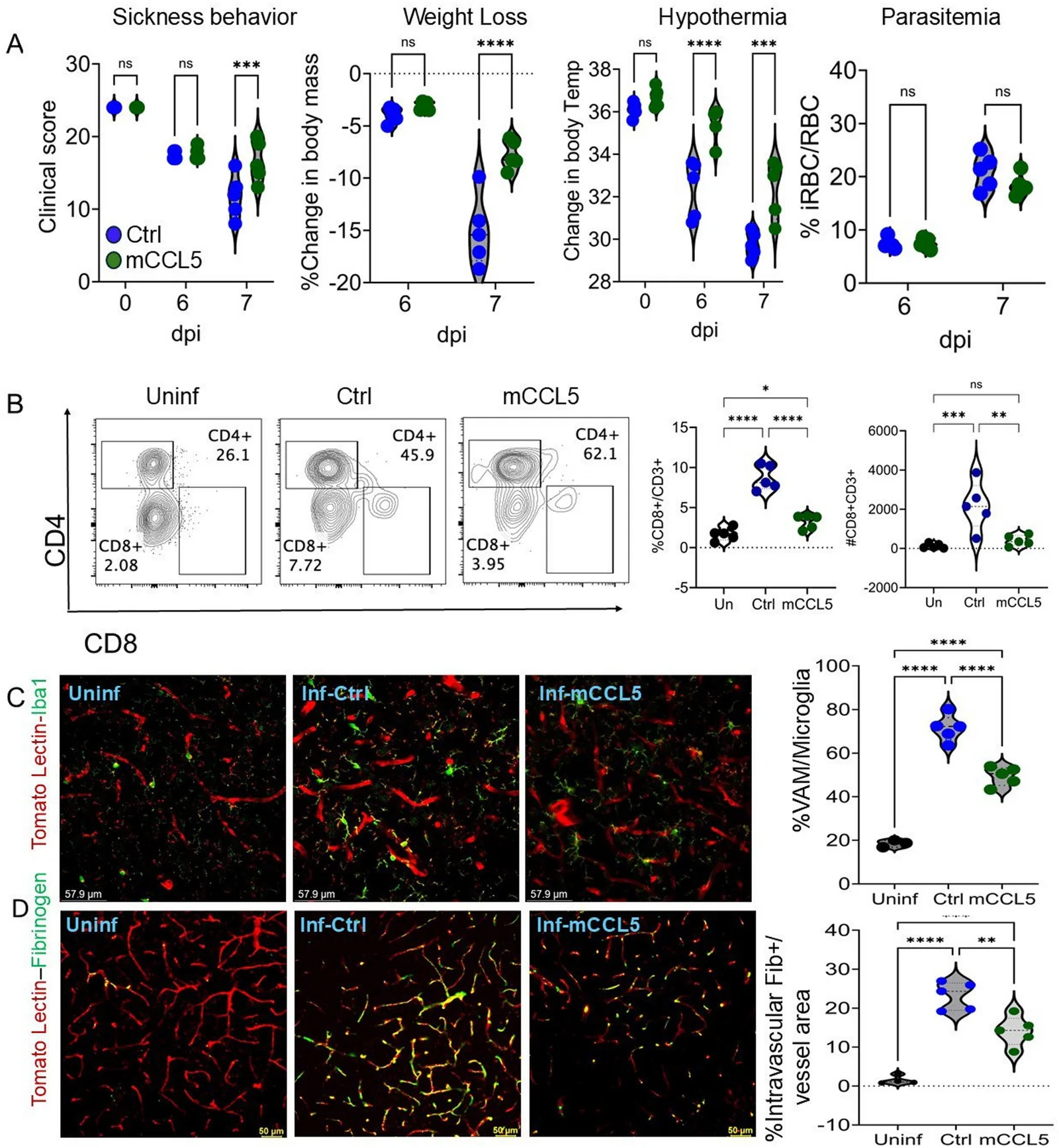
Inhibition of CCL5 chemokine receptor binding decreases sickness behavior, vascular-associated microglia, CD8 T cells, and fibrin(ogen) deposition in the brain but not parasitemia. IL-10 KO mice were infected with *P. chabaudi* and treated with met-CCL5 intranasally from 3 to 6 dpi. **A** SHIRPA Behavioral assay: percent change of body mass, temperature, and parasitemia (%iRBC/RBC) are shown as violin plots with all mice showing all animals and the range of 95% CI. **B** Contour plots show flow cytometry of Thy1^+^ brain cells and a violin plot showing quantification of percent CD8 + out of all T cells (CD45^hi^CD11b^−^Thy1^+^) in the brain. **C**,** D** Confocal micrographs showing immunohistochemistry of brain vessels stained with Tomato lectin (endothelial cells, red, Dylight 594) with (**C**) Iba1 or (**D**) fibrinogen (Green). **C** Vascular-associated microglia were quantified as the percentage of microglia with soma in direct contact with lectin-stained vessels. **D** Intravascular fibrinogen quantified as percent of lectin-stained vessels containing fibrin(ogen) (yellow). Data are represented as mean ± SEM. *p < 0.05, **p < 0.01, ***p < 0.001, ns, not significant as tested by ANOVA (**A**, **C**, **D**) and unpaired Student’s *t-test* (**A-D**)

Chemokines are critical in neuroinflammation during eCM, contributing to immune cell adhesion within the vasculature [46, 47]. C-C chemokine receptor (CCR)5, CCR2, and C-X-C- chemokine ligands (CXCL)-9, -10 (which use CXCR3) participate in cellular recruitment during eCM in *P. berghei* ANKA infected B6 mice [48–52]. We previously reported an increase in CCL5 protein in the brain vessels of *P. chabaudi*-infected- in comparison to uninfected IL-10 KO mice, where CCL5 colocalized in vascular areas with fibrinogen deposition and vascular-associated microglia [22]. Here, we assessed the role of CCL5-receptor signaling on microglial migration to the blood-brain barrier (BBB) in IL-10 KO mice infected with *P. chabaudi* using an antagonist of CCL5, Methionine-CCL5 (Met-CCL5) [38]. Intranasal treatment with Met-CCL5 from 3 to 6 dpi mitigated sickness behavior as measured by behavioral scoring between 0 and 7 dpi (Fig. 3A). Similarly, we observed less severe weight loss and hypothermia in IL-10 KO infected mice treated with the CCL5 antagonist vs. infected mice without treatment. No significant difference was seen in parasitemia, indicating that the difference observed is attributable to the effect of the inhibition of CCL5 chemokine receptors (binds to CCR5 > CCR1/2). Furthermore, flow cytometric analysis demonstrated a notable reduction in frequency and number of brain-infiltrating T cells, specifically CD8⁺ T cells, in Met-CCL5-treated animals (Fig. 3B), but not inflammatory monocytes (Supplemental Fig. 4A) or CD4 T cells (Supplemental Fig. 4C). As hypothesized, blocking CCL5 signaling intranasally resulted in a marked reduction in vascular-associated microglia (VAM), revealed by Iba1 immunostaining with Tomato lectin to label brain vasculature (Fig. 3C). Intravascular coagulation was also reduced by Met-CCL5 (Fig. 3D). There was no change in the serum cytokines TNF and IFN-*γ* (Supplemental Fig. 4C), supporting parasitemia data in indicating a limited systemic effect of intranasal Met-CCL5 treatment focused in the brain as shown [53]. These findings suggest that CCL5 chemokine receptors contribute to the recruitment of microglia to the vasculature and to the accumulation of cytotoxic T lymphocytes in the brain, which are known to exacerbate neuroimmune pathology during malaria infection [54, 55].

### The fibrin *γ*^*377–395*^ peptide reduces microgliosis and increases hypothermia during hyperinflammatory *e*CM

**Fig. 4:**
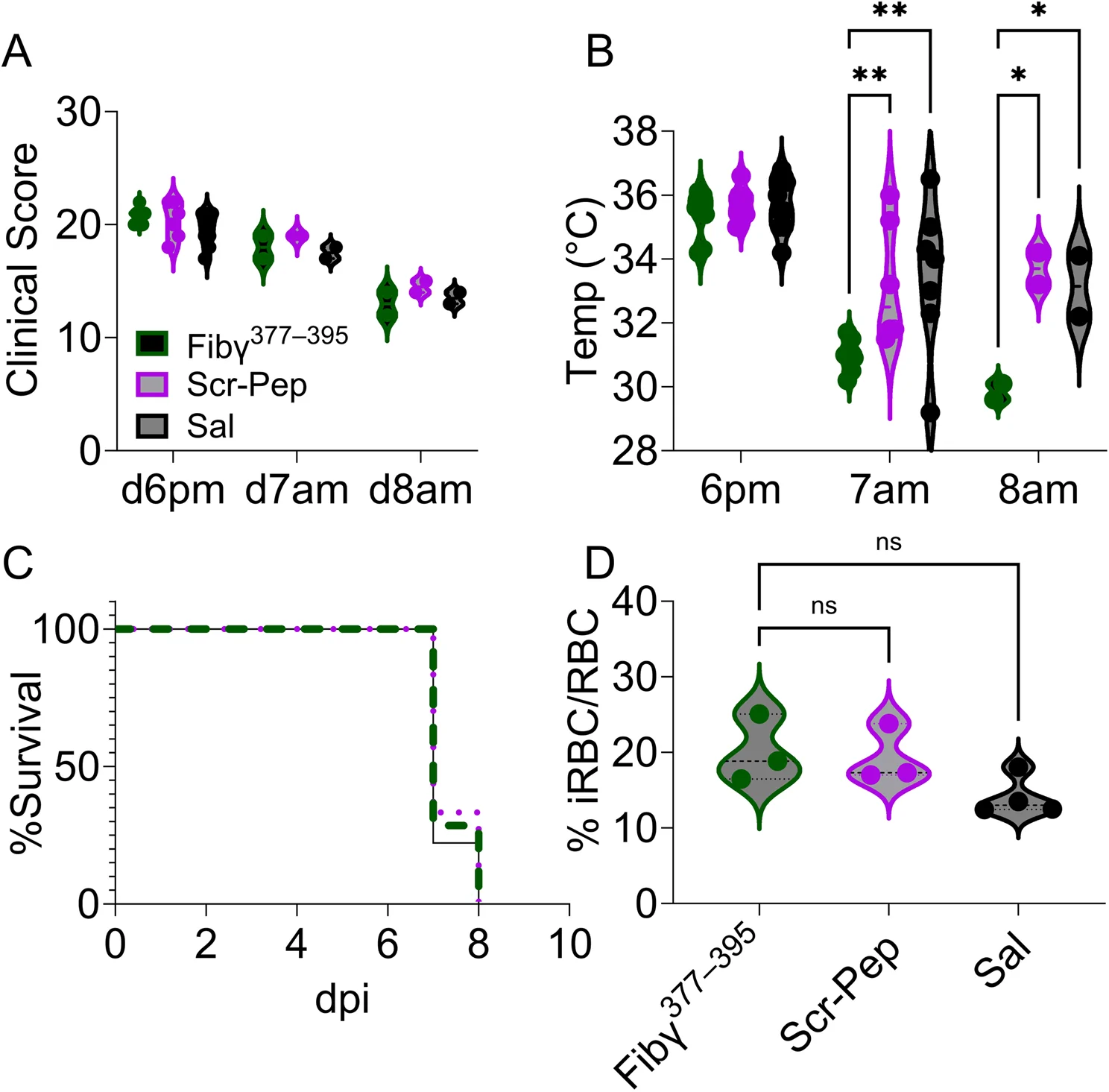
Peptide inhibitor of the fibrin-CD11b interaction increases hypothermia during hyperinflammatory *eCM. *IL-10 KO mice were infected with *P. chabaudi* and treated with *Fibγ*^*377–395*^, scrambled peptide, or saline from 4 to 8 dpi. Pathology was monitored daily to generate violin plots of (**A**) SHIRPA behavioral score, **B** body temperature, **C** survival, and (**D**) parasitemia (%iRBC/RBC). Data are presented as violin plots (95% CI) showing all animals and the range of 95% CI. Statistical analysis was performed with (**C**) Log-rank (Mantel-Cox) test, and (**A**,** B**) 2-way or (**D**) 1-way ANOVA, followed by Dunnett’s multiple comparisons test for significance versus saline control. The experiment was repeated twice with similar results (*n* = 5 per group). *p ≦ 0.05, **p < 0.01, ns, not significant

To test the relevance of the interaction between fibrin(ogen) and CD11b in eCM, specifically in the brain, we investigated whether a blocking peptide given intranasally would inhibit the activation of microglia by fibrin and affect the outcome of eCM. IL-10 KO mice were infected with *P. chabaudi* and treated with Fibrin γ^377–395^, a scrambled peptide, or saline daily from 4 to 8 dpi. Behavioral symptoms were assessed using the modified SHIRPA protocol (Fig. 4A). All experimental groups demonstrated significant deterioration in behavioral scores by 7 dpi. Animals treated with Fibrin γ^*377–395*^ peptide had significantly lower body temperatures than animals treated with control peptide or saline (Fig. 4B), consistent with the results of the Fibγ^390–396A^ mutant animals. Survival was comparable among the groups (Fig. 4C), and no differences were observed in parasitemia (Fig. 4D).

**Fig. 5:**
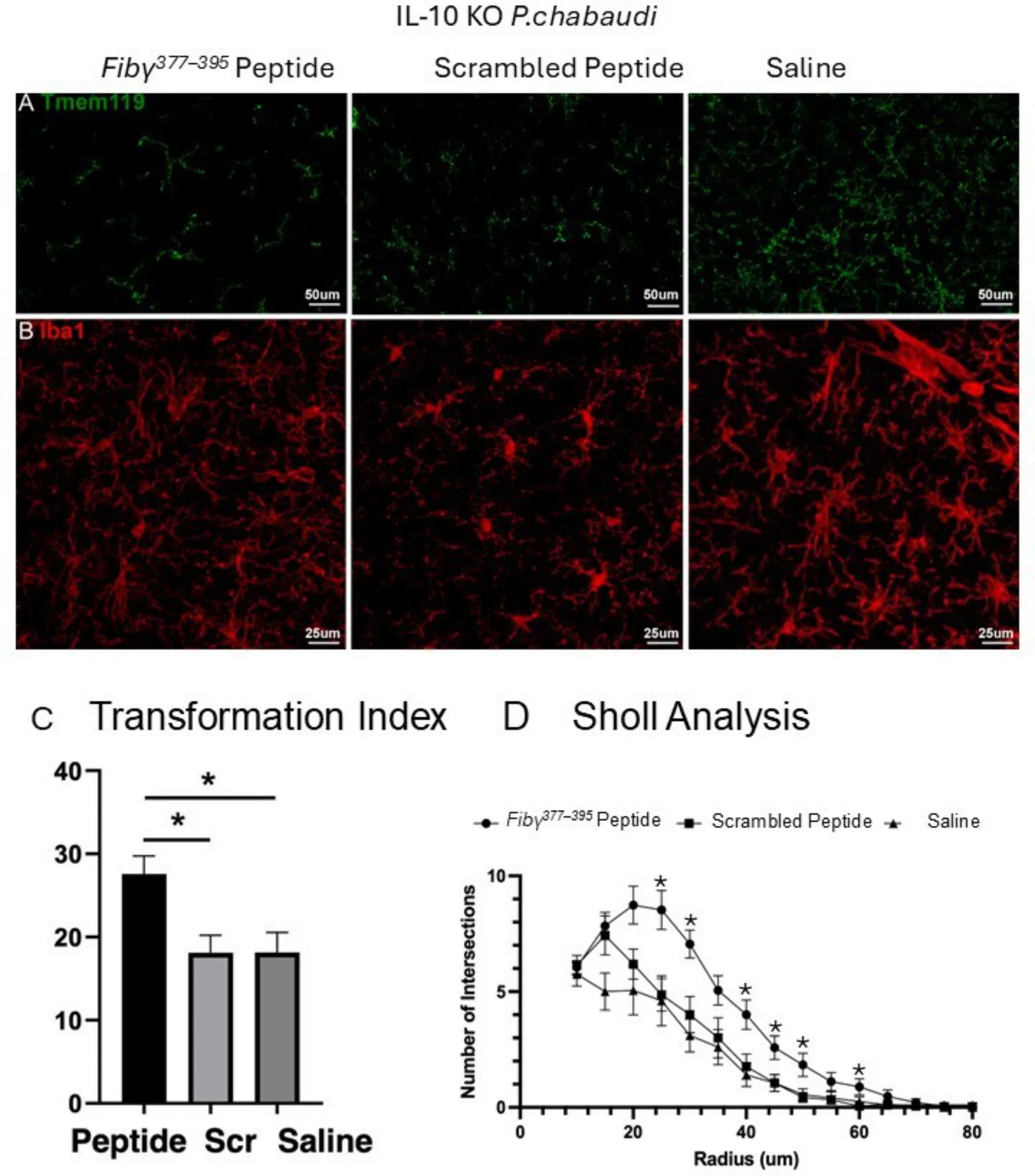
The fibrin γ377–395 peptide reduces microgliosis during hyperinflammatory eCM. IL-10 KO mice were infected with P. chabaudi and treated intranasally with fibrin γ^377–395^ peptide, scrambled peptide, or saline from 4 to 8 dpi. Immunohistochemistry staining with **A**) Tmem119 (Green) or **B**) Iba1 (Red). Quantification of microglial morphology using **C**) transformation index, represented as mean ± SEM and analyzed with one-way ANOVA between peptide and scrambled with posthoc Tukey’s; and **D**) Sholl analysis, which quantifies the length and number of microglial processes retracted upon activation. *p ≦ 0.05

The fibrin*γ*^*377–395*^ peptide decreases microglia activation in the EAE model of multiple sclerosis and models of stroke [56]. We tested the effect of the fibrin *γ*^*377–395*^ peptide in eCM by microglia marker and quantification. Immunostaining revealed that mice treated with the Fibγ^390–396 A^ peptide exhibited less microglia activation, both when stained with Tmem119 (Fig. 5A) and Iba1 (Fig. 5B) in comparison to the scrambled peptide and saline groups. The number and length of processes were used to quantify Iba-1-based morphology to determine the degree of activation of microglia using a transformation Index (Fig. 5C) and Sholl Analysis (Fig. 5D). Notably, a lower index of transformation or an increased number of intersections indicates that microglia have fewer or shorter cellular processes, suggesting a more activated state that is less like the homeostatic surveillant state [57]. Collectively, genetic and inhibitor data above suggest a protective role for Fibrin-CD11b interactions against hyperthermia in eCM that is also supported by microglia depletion studies [22].

### Genetic blockade of the fibrin-CD11b interaction increases hypothermia in *eCM*

**Fig. 6:**
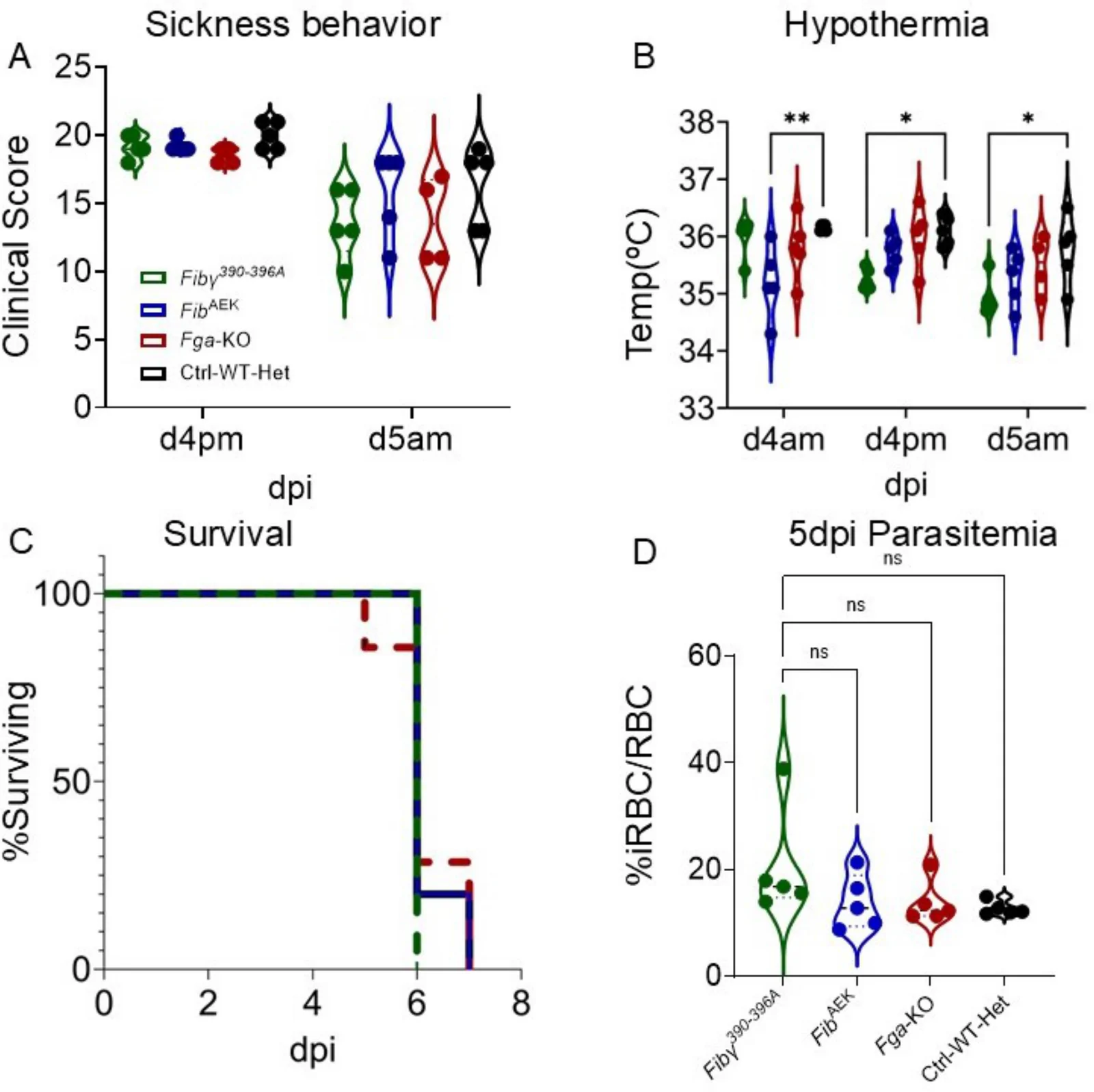
Genetic blockade of the fibrin-CD11b interaction increases hypothermia in *eCM. *Groups of fibrinogen mutant (Fibg^390–396A^, FibAEK, Fga KO) and wild-type mice (WT, Het) were infected with P. berghei ANKA. Pathology was monitored daily to generate violin plots of (**A**) SHIRPA behavioral score, **B** Body temperature showing hypothermia, **C** Survival curve, and (**D**) Parasitemia (%iRBC/RBC). Data are presented as violin plots showing all animals and the range of 95% CI. The survival curve was analyzed with the Log-rank (Mantel-Cox) test (**C**), while **A**, **B** use 2-way, and D 1-way ANOVA, followed by Tukey’s multiple comparisons test. The experiment was repeated two times with similar results (*n* = 5 per group). *p ≦ 0.05, **p < 0.01, ns, not significant

Fibrinogen not only serves as a key coagulation factor but also regulates inflammatory responses through interactions with immune cells. To investigate whether fibrinogen contributes directly to the development of cerebral malaria pathology, we utilized fibrinogen mutant mice in the *P. berghei* ANKA model of eCM. This approach allowed us to dissect the specific role of fibrinogen-mediated pathways in neuroinflammation and assess disease outcomes. We previously reported that microgliosis was dampened in IL-10 KO infected with *P. chabaudi* upon anticoagulant treatment [16] and that microglial depletion increased intravascular fibrin(ogen) deposition [22]. These data suggest a linkage between vascular leakage and neuroinflammation, as published previously in [56], and a role for fibrinogen in microglia activation, as has been reported in EAE [56] and Alzheimer’s disease [23]. To investigate whether deposition of fibrin(ogen) and vascular dysfunction or interaction with CD11b promotes neuroinflammation in eCM, we infected three types of fibrinogen mutant animals on the C57BL/6J (WT, B6) background with *P. berghei* ANKA, a highly pathogenic parasite infection that causes experimental cerebral malaria in this background. To distinguish the role of fibrin(ogen) in clotting from that in microglial activation, we included *Fib*^AEK^ mice, which make fibrinogen heterotrimers that can interact with platelets, but cannot be cleaved to make fibrin polymers; Fibγ^390–396A^mutant mice, in which fibrin(ogen) binding to CD11b is affected; and fibrinogen a deficient animals (*Fga* KO), which lack fibrinogen, limiting both clotting and CD11b binding [32, 56, 58]. In this experiment, fibrinogen mutant mice (*Fib*^AEK^, *Fga* KO, Fibγ^390–396A^) and a control group (C57Bl/6, Het) were infected with *P. berghei* ANKA. Behavioral and neurological symptoms were monitored using the abbreviated SHIRPA (Fig. 6A). There was no difference in the clinical score between groups, suggesting that the overall course of the disease is not affected differently across the groups. The mice that carried a mutation on the gamma chain of the fibrinogen gene (*Fibg*^*390–396A*^) showed a profound decrease in temperature compared to control mice (*p* = 0.0156) at 4 dpi PM and 5 dpi (*p* = 0.019) (Fig. 6B). *Fib*^AEK^ also showed an early decrease in temperature compared to control mice at 4 dpi (*p* = 0.0045). The survival curve shows no significant differences between fibrinogen-mutated and control mice, with all Fibγ^390–396A^ mice succumbing to cerebral malaria by 6 dpi. (Fig. 6C). Parasitemia was similar in all groups (Fig. 6D). Due to the difference between *Fib*^*AEK*^ and *Fga* KO, to further decrease coagulation in the *Fga* KO animals, we depleted platelets using anti-GPIb alpha on days 3, 5, and 6 dpi (Supplemental Fig. 5). However, there was no change in the disease course in this experiment. *Fibg*^*390–396A*^ showed increased hypothermia over controls, similarly to our previous observation in hyperinflammatory eCM in the fibrin γ^*377****–****395*^ peptide experiment.

## Discussion

In acute inflammatory disease, fibrinogen plays a dual role in pathology being critical to control blood loss following tissue damage and as part of the immune response by serving as an activation ligand of specific receptors on immune cells. This study, in the context of prior research, shows that both functions of fibrinogen contribute to the regulation of pathogenesis in hyperinflammatory severe malaria. Overall, interactions between microglia and fibrinogen regulate microgliosis and drive coagulation activation and fibrin-driven hemostatic function during experimental cerebral malaria. Strikingly, and not previously appreciated, fibrin(ogen)-dependent microgliosis following infection influenced the development of hypothermia during CM. The specific nature of sensing and phagocytosis of fibrinogen by microglia in eCM suggests that these cells act as sentinels for BBB, as in other neuropathologies [23, 24, 59]. Fibrinogen was primarily seen in microglia physically aligned with the vessels. Microglia are recruited to vessels containing fibrin deposits that have T cells and inflammatory monocytes contained within loci of the same vessels [16, 22].

Previously, we reported an increase in intravascular CCL5 expression in vessels harboring fibrinogen clots with microglia nearby, leading us to explore mechanisms of microglia migration. Here, we uncovered a dual role for the CCL5-signaling pathway in the recruitment of both microglia and adhesion of T cells. Several chemokines have been reported to be critical for eCM infection [60]. CXCL10 is strongly induced on the brain endothelium as early as 4 days after infection, while CXCL9 and CXCL10 expression are found in inflammatory monocytes within the blood vasculature on day 8 [52]. Genetic deficiencies in CCR2 [61] do not protect against eCM. This contrasts with other CNS inflammatory diseases, such as experimental autoimmune encephalomyelitis (EAE), where CCR1 and CCR2 are pathogenic drivers while CXCR3 and CCR5 are dispensable [62, 63]. CCR5 deficiency confers slightly less protection to *P. berghei* ANKA eCM than CXCR3 deficiency and limits CCR5⁺ CD8⁺ T-cell and monocyte accumulation in the brain and cerebrovascular leakage [64–66]. CCL5 RNA was reported to be present in infiltrating lymphocytes [67]. Of note, CCL5 and its receptor CCR5 have been implicated in microglia recruitment in other neuroinflammatory models [25, 26]. Notably, intranasal inhibition of CCL5 in *P. chabaudi*-infected IL-10 KO mice improved neurological outcomes and reduced neuropathology without altering parasitemia. Additionally, the intranasal route did not affect the peripheral immune response, as validated by serum cytokines. Together, these data suggest that the CCL5–CCR5 axis orchestrates both microglial migration to vascular sites of coagulation and T cell surveillance within the vessels, but CCR5 may also have an overall inflammatory contribution to eCM pathogenesis.

Notably, pro-inflammatory cytokines such as TNF are known to activate brain endothelial cells and upregulate ICAM-1 [68]. However, no effect of ICAM-1 on immune cell adhesion in the brain was detectable in our studies. ICAM-1-deficient animals do not get eCM from *P. berghei* ANKA infection in some studies [68] and, in some reports, have reduced recruitment of CD8 T cells [8]. An alternative explanation for the requirement of ICAM-1 in *P. berghei* ANKA eCM is that ICAM-1 contributes to platelet binding, a component of coagulation [69], or T cell activation [70]. However, platelets are also reported to kill the parasite [71]. In *P. chabaudi* infected ICAM- 1-deficient (IL-10 sufficient) animals, there was less parasite adhesion and higher peripheral blood parasitemia with less anemia and weight loss [45]. We cannot exclude the contribution of alternative entry routes such as skull bone marrow channels [72]. The negative results regarding the classical adhesion molecules suggest that if there is indeed tight adhesion rather than rolling, which can be mediated by selectins, there is a non-canonical mechanism of cellular recruitment in hyperinflammatory eCM. Resolving that conundrum, the current literature on *P. berghei* ANKA infection suggests that leukocyte adhesion via the CXCR3 ligand CXCL10 and MHCI are the main determinants of CD8 T cell activation and accumulation in the brain [52, 55]. Therefore, the literature suggests that IFN-γ-induced chemokines and antigen presented by the endothelium, rather than firm adhesion by classical adhesion molecules, really drive T cell accumulation in *P. berghei* ANKA infection [52, 72]. 

Microglia depletion in IL-10 KO mice infected with *P. chabaudi* led to a striking increase in fibrinogen deposition in the brain, implicating microglia in controlling inflammation-induced fibrin(ogen) accumulation, which is linked to early eCM mortality [22]. The presence of fibrin(ogen) within activated microglia and the regulatory role of microglia in coagulation suggest a potentially direct mechanistic link between microglia and the regulation of coagulation through phagocytosis that will need to be tested [16]. These findings are consistent with the mechanistic coupling of coagulation and microglia reported in neuroinflammatory disease [73]. In addition to the association of microglia with vessels containing coagula and their reduced activation upon anticoagulant treatment, this also suggested the possibility of a specific role for fibrinogen in microglial activation in eCM. Fibrin activates microglia via a CD11b-dependent interaction and contributes to local inflammation in multiple neuroinflammatory and neurodegenerative models [59]. However, this mechanism had not been thoroughly investigated in infectious disease models such as malaria. Prasad et al. reported impaired clearance and greater mortality from Staphylococcal peritonitis in Fib^AEK^ animals, suggesting a requirement for fibrin formation in clearing that bacterial infection [32]. Ryu et al. reported protection of the *Fga* KO and *Fibg*^*390–396A*^ mice in models of COVID-19 [74]. Despite a strong protective effect of the anticoagulant, LMWH, in hyperinflammatory eCM, they were little effect in fibrinogen mutants in *P. berghei* ANKA infection. Clinical scores and survival were unaffected by eliminating fibrinogen or blocking the interaction of Fibrin(ogen) with CD11b in hyperinflammatory eCM. The lack of a detectable phenotype in *Fga* KO mice may suggest an improvement from less coagulation, balanced by a negative effect on protective microglia; or reflect the delicate balance between hypercoagulation driving hemorrhage and some coagulation promoting vascular healing; or may suggest predominance of other aspects of the coagulation cascade in this model. Two other factors promoting coagulation have also been implicated in CM. High molecular weight von Willebrand factor multimers are also increased in human and mouse infection [75], and vWF deficiency delays vascular permeability and death in C57Bl/6 animals infected with *P. berghei* ANKA [76]. Tissue Factor is increased in infected individuals, and lower levels of Tissue Factor, but not complete deficiency, protect *P. berghei* ANKA-infected animals from microhemorrhage and early mortality. The carefully titrated balance of protection and damage by coagulation factors may also explain the lack of phenotype in AEK mice, which do not generate stable occlusive thrombi following FeCl3 injury of carotid arteries.

Reduced microglial activation was detected as decreased Iba1 and Tmem119 staining and morphological measures of activation. Similarly, previous findings established that fibrinogen leakage into the CNS induces microglial activation through binding to the CD11b/CD18 (Mac-1) integrin, promoting oxidative stress, synapse elimination, and neuronal injury in models of multiple sclerosis and Alzheimer’s disease [23, 24, 56, 77]. While microglia are overall more protective than inflammatory in hyperinflammatory eCM, our results here extend evidence of the detection of BBB breakdown by microglia through the interaction of CD11b and fibrin(ogen). Prior studies support our finding that microgliosis is linked to protection of animals from hypothermia in severe malaria [22]. IL-10-deficient animals depleted of microglia before infection died one day earlier of *P. chabaudi* infection with significantly more severe hypothermia. Our findings of more severe hypothermia in *Fibγ*^*390–396A*^ mice as well as exacerbated hypothermia in infected IL-10-deficient mice treated with the *Fibrin*^*377–395*^ peptide suggest this modulation of hypothermia is mediated by microglia binding of fibrin deposits within inflammatory foci. However, specific mechanisms linking fibrin-microglial interactions to modulation of body temperature in CM remain to be established.

This study further solidifies knowledge of the mechanistic links between cerebrovascular patency and microglial activation and supports growing evidence of a protective role of microglia in thermoregulation in eCM.

## Supplementary Information

Supplementary Material 1: Supplemental Figure 1: CX3CR1^GFP^ reporter microglia (green) uptake Fibrinogen-AF647 (Red). IL-10 KO CX3CR1^GFP^ CCR2^RFP^ reporter mice were infected with *P. chabaudi*. Fibrinogen-AF647 was given i.v. at 48 and 24 hours before imaging. Brains were imaged at 7 dpi using intravital microscopy by two-photon fluorescence microscopy through a thinned-skull cranial window. Video exhibits (CCR2^RFP+,^ red) adherent and rolling leukocytes along the vessel walls (intraluminal) and hypertrophic microglia (CX3CR1^GFP+^) in apposition to the outside of vessels, exhibiting uptake of fluorescent fibrinogen (Red). Flow cytometry (in Fig 1D) confirms that microglia were CX3CR1+ CCR2- with many CCR2+ leukocytes, both T cells and monocytes. Extensive intravital microscopy [22] supports that the red signal within microglia is due to uptake of AF647-fibrinogen, as in Supplemental 2.

Supplemental Material 2: CX3CR1^GFP^ reporter microglia uptake Fibrinogen-AF647. IL-10 KO CX3CR1^GFP^ CCR2^RFP^ reporter mice were infected with P. *chabaudi*, and Fibrinogen-Alexa 647 was given i.v. at 48 and 24 hours before imaging. Brains were imaged at 7 dpi using intravital 2-photon imaging. Video exhibits uptake of fluorescent fibrin(ogen) (AF647, Red) by hypertrophic activated CX3CR1^GFP^+ microglia (Green).

Supplementary Material 3: Supplemental Figure 3: Gating Strategy used in Figure 2. IL-10 KO mice were infected with *P. chabaudi*, and a single cell suspension of the brain was prepared 7 dpi. Forward and Side Scatter characteristics and live-dead staining are used to gate on single live cells. Microglia are defined as CD11b+CD45int, inflammatory monocytes are defined as CD11bhiCD45hi, and Ly6ChiMHCII+. T cells are defined by their CD45hiCD90(Thy1.2+) phenotype and then distinguished as CD4 or CD8.

Supplementary Material 4: Supplemental Figure 4: Intranasal inhibition of CCL5 did not change the fraction of adherent inflammatory monocytes (iMO) in the brain, nor CD4+ T cells, nor systemic T cell cytokines (TNF, IFN-γ ). IL-10 KO mice were infected with P. chabaudi, and mice were treated with Met-CCL5 from 3 to 6 dpi, while the control group was treated with saline. Brains were collected for flow cytometry at 7 dpi. Blood was also collected for cytokine measurement by immuno-bead assay. A) Graph of adherent inflammatory monocytes (CD11b+ CD45hi Ly6C+ MHCII+) and MFI of MHCII on inflammatory monocytes (iMO). B) Graph of %CD4+/Thy1.2+ T cells. C) Graph shows systemic cytokines TNF and IFN-γ; as an indication of systemic T cell activation. Statistical analyses were performed using an unpaired Student’s t-test; p-value not significant, ns.

Supplementary Material 5: Supplemental Figure 5: Platelet depletion in fibrinogen-deficient animals does not change eCM symptoms. Fga KO mice were infected with 10^6 P. berghei ANKA iRBC followed by anti-mouse GPIb(α) (CD42b) or isotype control to deplete platelets (3, 5, and 6 dpi, i.p.). A) SHIRPA behavioral clinical score, B) Weight change (% change), C) Survival curve, D) Parasitemia (iRBC/RBC). Data are presented as the mean with 95% CI, and were tested using 2-way ANOVA, followed by the Sidak multiple comparisons test, except for survival curves, analyzed using the Log-rank (Mantel-Cox) test. ns, not significant.

## Data Availability

The data that support the findings of this study are available from the corresponding author upon request.

## Abbreviations

CM: Cerebral malaria
eCM: Experimental cerebral malaria
MetCCL5: Methionine CCL5
TNF: Tumor Necrosis Factor
iRBCs: Infected red blood cell
i.p.: Intraperitoneal
i.v.: Intravenous
PBS: Phosphate-buffered saline
PFA: Paraformaldehyde
ºC: Degrees Celsius
dpi: Day post-infection
CNS: Central nervous system
Fib: Fibrinogen
Fga: Fibrinogen Alpha
Fibγ^390–396A^: Fibrinogen gamma
Fib^AEK^: Fibrinogen AEK
